# Perspectives in melanoma: Meeting report from the Melanoma Bridge (30 November–2 December, 2017, Naples, Italy)

**DOI:** 10.1186/s12967-018-1568-6

**Published:** 2018-07-21

**Authors:** Paolo A. Ascierto, Igor Puzanov, Sanjiv S. Agarwala, Carlo Bifulco, Gerardo Botti, Corrado Caracò, Gennaro Ciliberto, Michael A. Davies, Reinhard Dummer, Soldano Ferrone, Thomas F. Gajewski, Claus Garbe, Jason J. Luke, Francesco M. Marincola, Giuseppe Masucci, Janice M. Mehnert, Nicola Mozzillo, Giuseppe Palmieri, Michael A. Postow, Stephen P. Schoenberger, Ena Wang, Magdalena Thurin

**Affiliations:** 1 Melanoma, Cancer Immunotherapy and Development Therapeutics Unit, Istituto Nazionale Tumori-IRCCS Fondazione “G. Pascale”, Via Mariano Semmola snc, 80131 Naples, NA Italy; 2 Department of Medicine, Roswell Park Comprehensive Cancer Center, Buffalo, NY USA; 3 Medical Oncology and Hematology, St. Luke’s University Hospital and Temple University, Bethlehem, PA USA; 4 Earle A. Chiles Research Institute, Robert W. Franz Cancer Research Center, Providence Portland Medical Center, Portland, OR USA; 5 Istituto Nazionale Tumori-Fondazione “G. Pascale”, Naples, Italy; 6 Division of Surgery of Melanoma and Skin Cancer, Istituto Nazionale Tumori–Fondazione “G.Pascale”, Naples, Italy; 7 “Regina Elena” National Cancer Institute, Rome, Italy; 8 Department of Melanoma Medical Oncology, Department of Systems Biology, University of Texas MD Anderson Cancer Center, Houston, TX USA; 9 Department of Dermatology, University of Zurich Hospital, Zurich, Switzerland; 10 Massachusetts General Hospital, Boston, MA USA; 11 Department of Pathology and Department of Medicine, Section of Hematology/Oncology, The University of Chicago Medicine, Chicago, IL USA; 12 Division of Dermatologic Oncology, Department of Dermatology, Eberhard Karls University, Tuebingen, Germany; 13 The University of Chicago Medicine, Chicago, IL USA; 14 Refuge Biotechnologies, Menlo Park, CA USA; 15 Department of Oncology-Pathology, Karolinska Institute, Stockholm, Sweden; 16 Developmental Therapeutics Program, Cancer Institute of New Jersey, New Brunswick, NJ USA; 17 Istituto Nazionale Tumori Fondazione G. Pascale, Naples, Italy; 18 Unit of Cancer Genetics, Institute of Biomolecular Chemistry, National Research Council, Sassari, Italy; 19 Memorial Sloan Kettering Cancer Center, New York, NY USA; 20 Weill Cornell Medical College, New York, NY USA; 21 La Jolla Institute for Allergy and Immunology, La Jolla, CA USA; 22 Immune Oncology Discovery and System Biology, AbbVie, Redwood City, CA USA; 23 Cancer Diagnosis Program, Division of Cancer Treatment and Diagnosis, NCI, NIH, Rockville, MD USA

**Keywords:** Melanoma, Immunotherapy, Target therapy, Biomarkers, Combination strategies

## Abstract

Metastatic melanoma represents a challenging clinical situation and, until relatively recently, there was an absence of effective treatment options. However, in 2011, the advanced melanoma treatment landscape was revolutionised with the approval of the anti-cytotoxic T-lymphocyte-associated protein-4 checkpoint inhibitor ipilimumab and the selective BRAF kinase inhibitor vemurafenib, both of which significantly improved overall survival. Since then, availability of new immunotherapies, especially the anti-programmed death-1 checkpoint inhibitors, as well as other targeted therapies, have further improved outcomes for patients with advanced melanoma. Seven years on from the first approval of these novel therapies, evidence for the use of various immune-based and targeted approaches is continuing to increase at a rapid rate. Improved understanding of the tumour microenvironment and tumour immuno-evasion strategies has resulted in different approaches to target and harness the immune response. These new immune-based approaches offer the opportunity for various approaches with distinct modes of action being used in combination with one another, as well as combined with other treatment modalities such as targeted therapy, electrochemotherapy and surgery. The increasing number of treatment options that are now available has resulted in a growing need to identify which patients will derive most benefit from which treatments. Much research is now focused on the identification of biomarkers that can be utilised to help select patients for treatment. These and other recent advances in the management of melanoma were the focus of discussions at the third Melanoma Bridge meeting (30 November–2 December, 2017, Naples, Italy), which is summarised in this report.

## Introduction

Although surgical resection plays a fundamental role as a curative approach in the initial stages of malignant melanoma when the disease is diagnosed early enough, many patients present with more advanced, unresectable disease. Metastatic melanoma represents a challenging problem and, until recently, there were no effective treatment options. A review of randomised trials in metastatic melanoma that were published up until 2006, reported a complete response rate of just 4.1% and a median overall survival (OS) of 7 months [1]. However, in 2011, the treatment landscape in advanced melanoma was revolutionised with the approval of the anti-cytotoxic T-lymphocyte-associated protein (CTLA)-4 checkpoint inhibitor ipilimumab and the BRAF-targeted monoclonal antibody vemurafenib. Both immunotherapy and targeted therapy significantly improved OS in phase III clinical trials [2, 3]. Since then, the approval of new immunotherapies, especially the anti-programmed death (PD)-1 checkpoint pathway inhibitors, PD1 and PD1-ligand (PD-L1), as well as targeted therapies, have further improved outcomes for patients with advanced melanoma.

Since the approval of the first of these novel therapies, evidence for the use of various immune-based and targeted approaches continues to increase. Improved understanding of the tumour microenvironment and tumour immuno-evasion strategies has resulted in different approaches to target and improve the anti-tumour immune response. These new immune-based approaches offer the opportunity for various drugs with distinct modes of action to be used in combination with one another, as well as with other treatment modalities such as targeted therapy, electrochemotherapy and surgery. The increasing number of treatment options that are now available has resulted in a growing need to identify patients who will derive most benefit from specific treatments. Much research is now focused on the identification of biomarkers that can be utilised to help select patients for treatment.

## A unifying model for cancer immune responsiveness

The clinical success of immune checkpoint blockade with anti-CTLA-4 and anti-PD-1 or PD-L1 inhibitors in melanoma has highlighted the clear survival benefit achieved with cancer immunotherapy, which distinguishes it from chemotherapy and even targeted therapy. However, the next challenge for immunotherapy is to extend its utility to a broader range of cancers, including those that appear to be immune-resistant. One way to achieve this goal is to adopt strategies that combine checkpoint inhibition with other treatments.

A complex myriad of models to explain immune resistance to checkpoint inhibitors has been suggested. However, analysis of data from The Cancer Genome Atlas (TCGA) has suggested that cancer cells go through a conserved evolutionary bottleneck and face a Two-Option Choice by which they evade immune recognition by the immune-competent host. This choice involves adopting either an oncogenic process devoid of immunogenic stimuli in which tumour growth is dependent upon a stepwise oncogenic mechanism that avoids immune recognition (immune-silent tumours) or showing an entropic biology prone to immune recognition (immune-active tumours) but with compensatory immunosuppression [4]. These two processes may result in a lack of response to checkpoint blockade through entirely distinct mechanisms. Immunotherapy agents including checkpoint inhibitors are only effective against immune-active tumours enriched with immune regulatory mechanisms. However, although an immune-active landscape is a prerequisite for immune responsiveness, it is not sufficient alone to predict immune response. This may be because immune regulatory mechanisms are closely correlated in expression with tumour inflammation signatures [e.g., interferon (IFN)-γ-induced Immunologic Constant of Rejection (ICR) and tumour inflammation signature (TIS) [5]], indicating that immune suppression goes hand-in-hand with immune activation.

Future efforts to overcome immunotherapy resistance need to consider the immune landscape that is being targeted. Immune-active tumours may benefit from combined immunotherapies that can overcome immunoregulatory mechanisms. However, immune-silent tumours may need priming to induce immunogenic cell death and promote the recruitment of innate and adaptive immune cells before they become suitable targets for treatment with checkpoint inhibitors.

## System biology in melanoma session

### The obesity paradox of melanoma

Obesity is associated with increased risk of several cancers, as well as worse outcomes. However, in some cancers, obesity may only be linked with worse outcomes in early-stage disease, whereas obese patients with later-stage disease have improved outcomes; this is the so-called “obesity paradox”.

In melanoma, obese patients with early-stage disease (largely clinically localised disease) have been reported to have worse OS and worse melanoma-specific survival (MSS) [6]. This correlation remained significant after adjustment for age, gender and stage, but not after adjusting for C-reactive protein (CRP). However, in a meta-analysis of randomised controlled trials of patients (n = 599) with stage IV melanoma treated with combined dabrafenib (a BRAF inhibitor) and trametinib (a MEK mitogen-activated protein kinase inhibitor), obesity was associated with improved outcomes [7]. Indeed, there was an inverse linear relationship between body mass index (BMI) and hazard ratio (HR), such that the higher the BMI, the better the survival. Obese patients had similar disease-stage, ECOG status and serum lactate dehydrogenase (LDH) as normal weight patients but did have more frequent use of concomitant aspirin, beta-blockers, anti-diabetic drugs (including metformin) and statins. However, further analyses showed that the impact of obesity on outcomes was independent of these medications. Similarly, obese melanoma patients treated with vemurafenib plus cobimetinib (a MEK inhibitor) showed improved OS and progression-free survival (PFS) compared to patients with a normal BMI [7]. Obese patients have also shown improved OS and PFS when treated with immunotherapy, both with PD-1 monotherapy and with ipilimumab plus dacarbazine. However, no association between obesity and improved survival was observed in melanoma patients receiving dacarbazine chemotherapy alone in two separate cohorts. In a multivariate analysis, BMI was predictive of response to targeted and immuno-therapy but was not a prognostic factor. With both targeted therapy and immunotherapy, outcomes were gender-specific with the survival advantage conferred by obesity driven by strong associations in men with no significant association between obesity and outcomes in women.

A key question is how the information that men with stage IV melanoma have better survival outcomes if they are obese rather than normal weight can be utilised. Firstly, it is important to ensure that BMI data are collected for patients. BMI may also be a relevant parameter to consider in clinical trial design and stratification of patients. The impact of obesity at stage III disease should also be investigated, along with its effect on other therapeutic regimens (e.g. combined PD-1/CTLA-4 blockade, flat-dosing). Further research into the association of obesity with outcomes at a molecular, metabolic and immune response level is required.

### Integrating tumour and host factors as coordinated biomarkers for immunotherapy

Most responders to immunotherapy have a T cell-inflamed tumour microenvironment phenotype which is characterised by increased chemokine production, intratumoural CD8^+^ T cells, a type I IFN signature and the presence of immune escape inhibitory pathways. The activity of anti-PD-1 therapy in patients with head and neck cancer, gastric cancer, and others is associated with a T cell- inflamed tumour microenvironment phenotype at baseline. This phenotype also displays PD-L1 expression, regulatory T cells (Tregs), and indoleamine-2,3-dioxygenase (IDO), which are all associated with increased CD8^+^ T cell infiltration and an immune gene signature. In contrast, non-T cell-inflamed tumours are characterised by low inflammatory signature and absent CD8^+^ T cells, and immune escape appears to be mediated by T cell exclusion.

Understanding the molecular mechanisms that underlie the presence or absence of this spontaneous anti-tumour T cell response should enable the development of therapeutic solutions for patients lacking T-cell infiltration. Multiple tumour and host-derived factors appear to impact on the generation of the T cell-inflamed tumour microenvironment phenotype. These include somatic differences at the level of tumour cells, such as mutational landscape, antigenic repertoire and distinct oncogene pathways that are activated in different patients; germline genetic differences at the host level, e.g., polymorphisms in immune regulatory genes; as well as environmental factors, such as the commensal microbiota, immunological/pathogen exposure and patient history.

One example of a distinct melanoma-cell-intrinsic oncogenic pathway that contributes to a lack of T-cell infiltration in melanoma involves activation of the WNT/β-catenin signalling pathway, which prevents the host anti-tumour immune response by a failure to recruit Batf3 dendritic cells (DCs) [8]. β-Catenin-expressing tumours are resistant to checkpoint blockade therapy. Adoptive transfer of tumour-specific T cells also has been shown to not control β-catenin-expressing tumours, by a mechanism linked to failed trafficking of effector T cells [9]. The recruitment of effector CD8^+^ T cells is dependent on CXCL9/10 production by Batf3 DCs, which are absent from β-catenin-expressing tumours. This indicates that the absence of CD103^+^ DCs within the tumour microenvironment resists the effector phase of an anti-tumour T cell response, contributing to immune escape.

Environmental factors may also contribute to differences in responses between patients. Direct administration of a *Bifidobacterium* mix to tumour-bearing mice improved tumour-specific immunity and response to anti-PD-L1 treatment [10]. Combination treatment of *Bifidobacterium* plus an anti-PD-1 antibody resulted in the near complete stopping of tumour outgrowth, with the effect mediated by augmented DC function and enhanced CD8^+^ T cell priming and accumulation in the tumour microenvironment. It has also been shown that patients with metastatic melanoma who respond to anti-PD-1 therapy have distinct microbiota, with *Bifidobacterium longum* being one species more abundant, compared with non-responders. In addition, anti-PD-L1 therapy is effective in germ-free mice that receive human microbiota from anti-PD-1 responder but not from non-responders. These data suggest that improved responses to cancer immunotherapy may be possible by manipulation of the microbiota.

Given the multiple potential biomarkers across various tissues and compartments, an integrated approach that employs machine learning algorithms to identify combinatorial patterns of biomarkers linked to anti-PD-1 efficacy could be a more informative than individual biomarkers. These machine learning approaches are being pursued to identify patterns and personalised mechanisms of resistance and should help maximize predictive biomarker efficacy, generate new hypotheses about mechanisms of effect, examine gene-environment interactions and help develop new therapies to expand therapeutic benefits.

### Rational combination immunotherapy based on gene expression profiling

Tumours can be profiled at baseline as either T cell inflamed or non-inflamed, with the T cell-inflamed tumour microenvironment correlating with the efficacy of T cell based immunotherapies. When gene expression analysis based on the T cell-inflamed phenotype was applied to The Cancer Genome Atlas a wide variation in frequency of T cell-inflamed samples was observed across tumour types, with the highest frequency in clear-cell kidney cancer and lung adenocarcinoma and the lowest in paraganglioma and low-grade glioma. In melanoma, no difference in multiple antigen classes was observed between T cell-inflamed and non-inflamed tumours [11]. There was also no correlation between gene expression and mutational burden in any cancer type, indicating that lack of spontaneous immune infiltration is unlikely to be caused by the lack of antigens.

Most therapeutic immune targets currently in clinical development show strong correlation with PD-L1 expression. However, the correlation between PD-L1 expression level and immunotherapy targets is weaker in non-inflamed tumours. Immune target genes can be separated into those that are strongly correlated with PD-L1 and those that are weakly correlated. For example, there is a strong correlation between high PD-L1 and high T cell immunoglobulin and mucin-domain containing-3 (TIM-3); almost all metastatic melanoma samples have either high PD-L1/high TIM-3 or low PD-L1/low TIM-3 expression. Other therapeutically relevant molecules in melanoma that PD-L1 is associated with include, but are not limited to, lymphocyte-activation gene (LAG)-3, IDO-1, forkhead box (FOX)P3, CTLA-4, colony stimulating factor 1 receptor (CSF1R) and glucocorticoid-induced tumour necrosis factor receptor (GITR). These targets tend to cluster in groups associated with varying levels of PD-L1 expression in melanoma as well as in other highly T cell inflamed tumours, such as head and neck cancer, lung adenocarcinoma, clear-cell kidney cancer and bladder cancer.

**Fig. 1:**
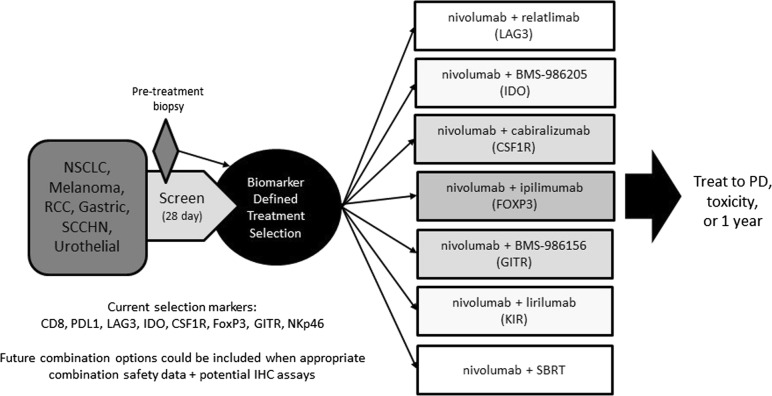
Adaptive biomarker trial design

Patient-level immune target identification may be feasible and may allow personalised immunotherapy. The Adaptive Biomarker Trial that Informs Evolution of Therapy after Nivolumab (ADVISE, NCT03335540) is designed to evaluate the treatment of solid tumours with various nivolumab (a fully human PD-1 immune-checkpoint-inhibitor antibody) based immunotherapy combinations, with treatment choice based on a broad biomarker assessment (Fig. 1).

Comprehensive tumour profiling of multiple tumours to characterize expression of lymphocyte-activation protein (LAG)-3 showed that it is moderately co-expressed with other immune markers and highly expressed on major histocompatibility complex (MHC) II high cells [12]. Increased tumour MHC II was observed in both inflamed and non-inflamed tumours and correlated with LAG-3-positive tumour-infiltrating lymphocytes (TILs). Preferential localisation of LAG-3-expressing leukocytes to MHC II high tumour regions potentially serves as a mechanism for LAG-3 checkpoint pathway activation. Tumours with high MHC II expression generally had lower PD-L1 expression. LAG-3 was upregulated during nivolumab monotherapy. The observation that nivolumab may induce LAG-3 expression highlights the need to define predictive biomarker profiles for the anti-LAG-3 agent, relatlimab, in PD-1-naïve and previously-treated patients.

## Biomarker session

### Biomarkers for checkpoint inhibition in melanoma: current knowledge and future directions

An important aspect of checkpoint inhibitor therapy are the durable responses that have been observed in patients who discontinue treatment. In the CheckMate-069 trial of combined nivolumab and ipilimumab, patients discontinuing treatment early due to drug toxicity had a high and durable response rate and derived an OS benefit that was comparable to that observed in the overall study population (18-month OS rate of 80% vs. 73%) [13]. This ‘first-shot’ theory suggests that response at ~ 3 months is an excellent marker of longer-term efficacy in checkpoint inhibition. Indeed, the outcome of ‘first-shot’ treatment may be the most important biomarker for long-term durable clinical success.

PD-L1 expression alone is not a valid selection criterion for treatment. In the CheckMate-067 trial, objective response rate (ORR) with nivolumab plus ipilimumab was increased in patients with PD-L1 tumour expression. However, when compared with ipilimumab alone, ORR was 15% higher in patients with PD-L1-positive tumours and 13% higher in patients with PD-L1-negative tumours. OS is also improved in patients with PD-L1 expression. However, PD-1 antibodies are still effective in patients without PD-L1 tumour expression. Murine double minute-2 (MDM2) amplification and hyper-progression under checkpoint inhibition may also be a biomarker of lack of efficacy. MDM2 is overexpressed in diverse tumour types and exerts its oncogenic effects primarily through inhibition of the p53 tumour suppressor protein.

The association between blood-based biomarkers and outcomes in patients receiving immunotherapy has also been widely investigated. In 209 patients with advanced melanoma treated with ipilimumab, low baseline LDH, low absolute monocyte counts, and low Lin^−^CD14^+^HLA^−^DR^−/low^-myeloid-derived suppressor cell (MDSC) frequencies were significantly associated with improved survival, as were high absolute eosinophil counts, high relative lymphocyte counts, and high CD4^+^CD25^+^FoxP3^+^-Treg frequencies [14]. Using a combined prognostic score consisting of absolute eosinophil and monocyte counts, relative lymphocyte counts and LDH, the number of favourable factors (4 vs. 3 vs. 2–0) was also associated with OS. Similarly, in patients treated with pembrolizumab (humanized monoclonal IgG4-kappa isotype antibody against PD-1), high relative eosinophil count and relative lymphocyte counts, low LDH, and absence of metastasis other than soft-tissue/lung were independent baseline characteristics associated with favourable OS; the presence of four favourable factors in combination identified a cohort with excellent prognosis [15]. A prognostic score based only on relative eosinophil and lymphocyte counts also identified patients most likely to have better OS. In another analysis, early increases in absolute lymphocyte counts (at 2–8 weeks after the first dose of ipilimumab) and delayed increases in CD4^+^ and CD8^+^ T cells (at 8–14 weeks) were correlated with improved survival [16]. Further investigation of these peripheral blood biomarkers is required. Established prognostic markers such as tumour stage and LDH are valid in checkpoint inhibitor therapy. However, it is uncertain whether lymphocyte and eosinophil counts are predictive or just prognostic markers.

Another possible predictive factor is functional T cell response. The presence of circulating T cells responding to peptides from Melan-A or NY-ESO-1 had strong independent prognostic impact on survival in patients with distant melanoma metastases [17]. Median OS of patients with responsive T cells was 21 months compared with 6 months for patients with non-responsive T cells. Patients with ≥ 2 targeted antigens also had significantly improved OS versus patients with 0–1 antigens. Barcode-labelled peptide-MHC multimers enable the combination of functional T cell analysis with large-scale epitope recognition profiling and allows the detection of low-frequency CD8 T cells specific for virus- or cancer-restricted antigens [18].

### FOLFIRINOX, immune response and clinical course of the disease in pancreatic ductal adenocarcinoma

It has been known for many years that malignant transformation of human cells may be associated with changes in the expression of HLA class I and HLA class II antigens. Defects in HLA class I antigen expression and/or function by tumour cells have a negative impact on their interactions with cognate T cells; as a result, they provide tumour cells with an escape mechanism from immune surveillance. The anti-tumour activity of chemotherapy and radiation is mediated, at least in part, by their ability to induce or enhance an anti-tumour immune response. In patients with pancreatic ductal adenocarcinoma (PDAC), the combination chemotherapy regimen FOLFIRINOX (oxaliplatin, irinotecan, fluorouracil, and leucovorin) is associated with improved PFS and OS compared with gemcitabine [19]. The beneficial effect of FOLFIRINOX on the clinical course of PDAC is in part mediated by the induction of changes that facilitate tumour cell recognition by the immune system. In patients with PDAC treated with neoadjuvant FOLFIRINOX, there is a reduced HLA-A defect frequency. In addition, there are significant increases in CD8^+^ T cells and Granzyme B^+^ cells and a significant decrease in FoxP3^+^ cell tumour infiltration density. A significant increase in CD4^+^ cell tumour infiltration density in patients treated with FOLFIRINOX and proton-beam therapy has also been observed, suggesting that chemotherapies have the ability to enhance tumour antigen-specific T cells.

### Towards precision immunotherapy of solid tumours: an HLA-agnostic functional neoantigen discovery platform

Advances in genomic sequencing and bioinformatics have led to numerous demonstrations of spontaneous and therapy-induced T cell responses against a subset of immunogenic tumour-specific somatic mutations referred to as neoantigens, raising the possibility that patients could be treated with vaccines personalised against the mutations expressed by their own tumour.

**Fig. 2:**
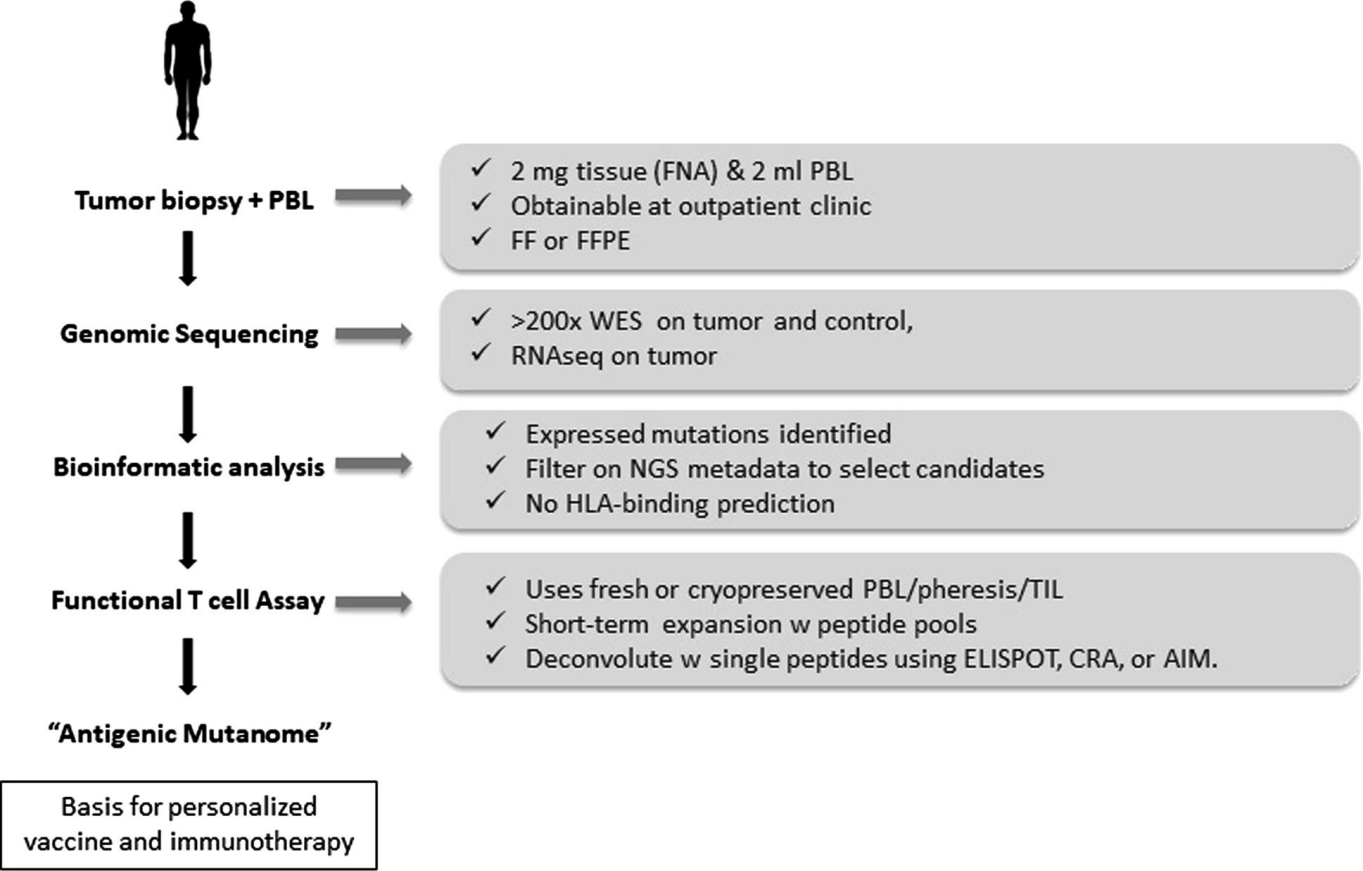
Neoantigen discovery and functional validation

Although neoantigens are targets for T cell recognition, their reliable discovery and validation remains a major challenge. Most strategies are based on modelling which peptides bind to the MHC-I molecule. Most neoepitope studies identify thousands of somatic mutations and predict a much smaller number of MHC binders. However, given that fewer than 0.3% of mutations can be confirmed as neoantigens, the chances for successful personalised immunotherapy might appear to be limited. At the San Diego Center for Precision Immunotherapy, a novel platform to identify neoantigens has been developed involving a combination of genomic sequencing, bioinformatic analysis, and functional testing of autologous PBMC or TILs (Fig. 2). This has minimal tissue and peripheral blood mononuclear cell requirements, detects both CD8^+^ and CD4^+^ T cell responses and can verify 20 to > 60% of selected mutations as neoantigens. The platform detects both driver and passenger mutations and can be used with low mutational burden tumours. Identifying a tumour-specific antigenic mutanome can be used as the basis for personalised immunotherapy and/or vaccination. To validate the neoantigen targets identified using this platform, we have developed a patient-derived xenograft (PDX) system through which neoantigen-specific TIL are used in adoptive immunotherapy against autologous tumours. These studies have shown a direct relationship between the frequency of neoantigen-specific T cells in a given cellular product and its therapeutic efficacy. These findings support the concept of truly personalized immunotherapy in which the specific neoantigens to be targeted are functionally validated on a per-patient basis.

### Valuable biomarkers to direct therapy: are we any closer?

Multiple potential biomarkers to predict the response to immunotherapy are likely to be needed given the complexity of the immune tumour microenvironment. However, the search for reliable biomarkers is limited by our incomplete understanding of how immunotherapies modify the already complex immune response to cancer. To be of clinical value, biomarkers need to be accurate, reproducible, minimally invasive, dependent on clinical situation and able to direct optimal selection and sequencing of cost-effective therapy. Current candidates include PD-L1 expression, CD8^+^ TILs, tumour mutation load, neoantigen burden and gene expression profile (GEP) (Table 1). PD-L1 tumour expression has been shown to correlate with response to anti-PD-1 antibodies; however, the lack of expression does not preclude a response. Variability in assays, antibodies, and tumour cell types detected as well as determining optimal cut-off points represent challenges in using PD-L1 expression as a biomarker. Additionally, PD-L1 expression is heterogenous and dynamic within an individual and can be induced by activated tumour-specific T cells; heterogeneity in PD-L1 expression is frequent within a sample as well as between the primary lesion and its metastases.

**Table 1:** Selected potential biomarkers for immunotherapy

	Basis	Challenges
PD-L1	IHC approach to measuring PD-L1 expression on tumour and immune cells	Variability in assays, antibodies and tumour microenvironment
CD8^+^ T cells	PD-1/PD-L1 expression on CD8^+^ T cells predicts response to PD-1 agents	Optimal cut-off points, scoring metrics and agreement on magnitude of change needed for meaningful prediction of response
Tumour mutation load	High mutation load resulting from various factors correlated with response to checkpoint inhibitors in exceptional responders	Availability of adequate tissue for sequencing; whole exome sequencing expensive and slow turnaround time vs. other clinical assays
Neoantigen burden	Predict clinical benefit to ipilimumab and PD-1 blockade in melanoma and lung cancer	As above
Gene expression profiling	IFN-induced signatures may predict response to checkpoint inhibitors	Sizable tissue collection needed to validate testing and training sets

The presence of Tregs and expression of PD-L1 and IDO are associated with a CD8^+^ cell infiltrate. T cell-inflamed tumours showed high expression of IDO, PD-L1, and FoxP3^+^ Tregs, suggesting that these inhibitory pathways might serve as negative feedback mechanisms that follow CD8^+^ T cell infiltration [20].

Tumour mutation burden (TMB) has been shown to correlate with response to checkpoint blockade, suggesting that the T cell response may be targeted to neoantigens that evolve as the mutation rate increases in the tumour cell, rather than established antigens. TMB may be as predictive as neoantigen load for response to immunotherapy. In a study using whole-exome sequencing of non-small-cell lung cancer treated with pembrolizumab, higher mutation burden in tumours and clinical responses were correlated with molecular signature characteristics of tobacco carcinogen-related mutagenesis, higher neoantigen burden, and DNA repair pathway mutations [21]. In 110 patients with melanoma, overall mutation load, neoantigen load and expression of immune microenvironment cytolytic markers were associated with clinical benefit, but no recurrent neoantigen peptide sequences predicted response to ipilimumab [22]. Mutational load, as determined by a next generation sequencing (NGS) platform available in the clinic may effectively stratify melanoma patients by likelihood of response to anti-PD-1 therapy [23].

Genomic assessment of exceptional responders may reveal patient groups that are extremely sensitive to checkpoint inhibitor therapy and can help inform different mechanisms of response within the same disease cohort. For example, genomic profiling of a pretreatment tumour sample from a patient with endometrial cancer who had an exceptional response to pembrolizumab identified a mutation in DNA polymerase epsilon (POLE) that was associated with an ultramutator phenotype [24]. Analysis of TCGA revealed that the presence of POLE mutation is associated with high TMB and increased expression of several immune checkpoint genes, indicating that cancers with POLE mutations are potential candidates for checkpoint blockade therapy.

TMB does not, however, always correlate with treatment response. This may be due to the poor dynamic range of the assays available or confounding by tumour purity, or because exome sequencing does not identify all types of mutations. The type of mutation may be as important as the number of mutations. In addition, immune activation may occur through processes that are independent of TMB, such as viral infection. Tumour samples from a patient with metastatic gastric cancer who responded to the anti-PD-L1 antibody avelumab showed no evidence of high mutation burden or mismatch repair defect. However, the tumour was strongly positive for presence of Epstein-Barr virus (EBV) encoded RNA and had evidence of immune infiltration, suggesting that EBV-positive low-mutation burden gastric cancers have immune activation and may respond to immune checkpoint therapy. Thus, there may be multiple mechanisms of immune activation, which may be very similar with regard to immunological signature [25].

While mutational load reflects tumour antigenicity, GEP reflects activated T cells in the tumour microenvironment. Analysis of GEPs using RNA from baseline tumour samples of pembrolizumab-treated patients resulted in the identification of an 18-gene immune-related signatures that was correlated with clinical benefit. This T cell-inflamed GEP contained IFN-γ-responsive genes related to antigen presentation, chemokine expression, cytotoxic activity and adaptive immune resistance and is currently being evaluated for potential diagnostic use in ongoing clinical trials [26]. In data from the KEYNOTE-028 trial in patients with solid tumours, both mutational load and GEP score were independently predictive of clinical response [27].

### How to establish precision medicine in metastatic melanoma

Multiplatform tissue processing can be used to help identify molecular characteristics of melanoma in order to help stratify patients for therapy. Whole exome sequencing (WES) can identify recurrent genomic aberrations, including somatic mutations in driver oncogenes (e.g. BRAF, NRAS, and KIT), tumour suppressor genes (e.g. CDKN2a, PTEN, and P53), inherited mutations in oncogenes, and the presence of known and new candidate mutations that cause treatment resistance.

A melanoma-specific gene panel, known as MelArray, has been developed based on the WES at USZ and Yale University. This panel is composed of 195 melanoma mutant genes [single-nucleotide variants (SNVs), copy-number variants (CNVs) and gene fusions] and has a sequencing cost that is approximately 20% lower than WES. It provides deeper coverage of important loci, empirical CNV measurements, gene fusions, non-coding variants, HLA type and T-cell receptor (TCR) sequencing. CNVs in the tumour are reported with a reference to the affected gene, deletion or amplification. Oncogenicity and mutation effects of CNVs and SNVs are reported in the oncogenic gene table, supported by literature references as evidence for the function of the oncogenic mutation or copy number alteration. Actionable genes are reported by matching oncogenic genes to the OncoKB database. OncoKB is an expert-guided precision oncology database that annotates the biological and oncogenic effect and the prognostic and predictive significance of somatic molecular alterations with the aim of supporting evidence-based treatment decision-making. To date, over 3000 unique mutations, fusions, and copy number alterations in 418 cancer-associated genes have been annotated. The oncogenic variant is listed with cancer type and corresponding treatment together with level of evidence. In an analysis of 83 melanoma samples, 70 had a potential drug for their specific oncogenic variant.

High-dimensional single-cell mass cytometry (CyTOF) can be used to characterise immune cell subsets in the peripheral blood of patients. Antibodies are labelled with metal isotopes rather than fluorochromes which avoids spectral overlap or autofluorescence problems. Simultaneous analysis of a large number of markers (> 50) is possible and barcoding allows analysis of multiple samples at the same time. Algorithm-guided analysis allows the clustering of cell subtypes. Assessment of changes in of responders versus non-responders before and after treatment allows for identification of potential biomarkers of response or the mechanism of the drug action. A strong predictor of response to anti-PD-1 treatment was the frequency of CD14^+^CD16^−^HLA^−^DR^hi^ monocytes [28]. Responders also had a lower frequency of circulating CD4^+^ T effector cells and CD8^+^ naïve T cells. In addition, numbers of multifunctional CD8^+^ cells expanded and CTLA-4 and Granzyme B expression levels were higher in responders. Several myeloid markers were upregulated in responders compared with non-responders. As such the frequency of monocytes in peripheral blood mononuclear cells may help in clinical decision-making regarding anti-PD-1 therapy.

## Combination strategy session

### How much better are anti-CTLA-4 plus anti-PD-1 combinations than anti-PD-1 alone

In the CheckMate-067 study of patients with advanced melanoma, combination therapy with nivolumab plus ipilimumab was associated with significantly longer OS than with ipilimumab alone [29]. Three-year OS was 58% with combined nivolumab plus ipilimumab, 52% with nivolumab alone and 34% with ipilimumab alone. On the basis of these findings, treatment with either combined anti-PD-1/anti-CTLA-4 therapy or PD-1 monotherapy appear to be reasonable options given the absence of a significant OS difference. However, it is possible that certain patient subgroups may derive more benefit from the combination than single agent anti-PD-1. In subgroup analysis, BRAF status, PD-L1 expression < 1% and region (US vs. EU) were the only factors that may be associated with OS benefit to the combination compared to single agent anti-PD-1, with region (US vs. EU) being the only significant factor in multivariate analysis [30]. The reasons for this are unclear as treatment exposure, management of adverse events, and use of subsequent therapies did not differ substantially between the two regions.

Further evidence on who to treat with combination therapy vs. anti-PD-1 alone is needed. Blood-based biomarkers [high LDH, low relative lymphocytes, low relative eosinophils, and presence of visceral (non-lung) metastases] have been associated with poor outcomes among patients treated with pembrolizumab [15]. Some of these factors have also been found to be associated with worse survival with nivolumab plus ipilimumab as well. An examination of these factors in the Checkmate 067 study is needed to see if they help distinguish which patients may benefit most from combination immunotherapy.

Patients with melanoma brain metastases are another group who might be considered for combination immunotherapy. In the Anti-PD-1 Brain Collaboration (ABC) study, intercranial response rate was higher in patients treated with combined nivolumab plus ipilimumab versus nivolumab alone [31]. Intercranial PFS was also improved with combination therapy (6-month median PFS rate of 46% vs. 28% with monotherapy).

Choice of combined anti-PD-1/anti-CTLA-4 therapy or PD-1 monotherapy may be influenced by patient factors, such as their resilience to increased early toxicities as well as their reliability in reporting any such side effects. Combination therapy may also be preferable for patients that need treatment with a higher response rate, given the lack of a clearly proven survival benefit. Newer dosing regimens of combination anti-CTLA-4 and anti-PD-1 are being tested to see if the high degree of efficacy of the current nivolumab + ipilimumab combination can be maintained with more favourable tolerability.

### Combining oncolytic therapy with checkpoint inhibitors

An immune-active tumour microenvironment and type I IFN transcriptional signature are associated with clinical benefit from immunotherapy suggesting that strategies targeting the type I IFN pathway could sensitize tumours to immune checkpoint blockade. Intralesional tumour therapy with oncolytic viruses results in type I IFN production and immunogenic cell death. Talimogene laherparepvec (T-VEC) is an oncolytic virus therapy based on a modified herpes simplex virus type-1 (HSV-1) that is designed to selectively replicate in tumours. T-VEC administration produces granulocyte–macrophage colony-stimulating factor (GM-CSF) and enhances local and systemic anti-tumour immune responses, causing regression in injected lesions and inducing immune responses that mediate regression at uninjected sites. In a phase III clinical trial, T-VEC was well tolerated and resulted in a higher durable response rate and longer median OS than subcutaneous GM-CSF administration [32]. In an open-label, multicentre trial, T-VEC in combination with ipilimumab, had a tolerable safety profile, ORR of 50%, 18-month PFS of 50% and 18-month OS of 67% [33]. Thus, the combination appeared to have improved efficacy compared with either T-VEC or ipilimumab monotherapy. Total frequency and activated CD8 T cells increase after T-VEC and after T-VEC plus ipilimumab. In the first randomised trial to evaluate addition of an oncolytic virus to a checkpoint inhibitor, ORR was significantly higher with T-VEC plus ipilimumab versus ipilimumab alone (39% vs. 18%; odds ratio 2.9; 95% CI 1.5–5.5; p = 0.002) [34]. With a median follow-up of 14.7 months, median PFS was 8.2 months (95% CI 4.2–21.5) in the combination and 6.4 (95% CI 3.2–16.5) with ipilimumab alone. The combination was tolerable with no unexpected safety findings with comparable gastrointestinal toxicity to the combination arm.

Another replication-competent oncolytic virus derived from HSV-1 is HF10, a spontaneously occurring HSV-1 mutant. A phase II trial of HF10 plus ipilimumab in patients with unresectable or metastatic melanoma demonstrated a favourable risk–benefit profile and encouraging antitumour activity, with best ORR at 24 weeks of 41% [35]. Treatment was well tolerated with no dose-limiting toxicities; most HF10-related adverse events were ≤ grade 2 and similar to those with HF10 monotherapy.

The combination of oncolytic virus and anti-PD-1 therapy may be an especially attractive option for patients who progress after first-line treatment with PD-1 agents and have injectable lesions, although this group of patients has not been extensively studied. In addition to T-VEC and HF10, several other oncolytic viruses are in development (e.g. Newcastle disease virus, Coxsackie virus) as well as other locally administered treatments and selection of the most appropriate therapy for individual patients becomes an increasing issue as treatment options expand. As such, biomarkers for selection are an important consideration.

### Combining electrochemotherapy and checkpoint inhibitors

Electrochemotherapy (ECT) is a tumour ablation modality that involves the local application of short duration high-voltage pulses in order to produce a transient increase in cell membrane permeability to cytotoxic chemotherapeutic drugs, such as bleomycin or cisplatin. ECT is reliable, effective and safe and can be used to treat (1) early cutaneous relapses after previous surgical treatment, (2) complete or partial progression after previous ECT treatment, (3) as palliative treatment of haemostatic or painful lesions, and (4) as neoadjuvant therapy for extensive lesions to reduce surgical approach. Several studies of ECT in patients with advanced melanoma have been reported and suggest good response rates and durable benefits. In a study of 60 patients with relapsed and refractory cutaneous melanoma metastases or in-transit disease who underwent 100 courses of ECT with intravenous injection of bleomycin, long-term durable benefits were achieved without a negative impact on quality of life [36]. ECT is inexpensive, simple to apply, well tolerated and appears to be an effective procedure for the local treatment of malignant tumour nodules, and objective responses under certain circumstances. However, there is no evidence that ECT alters the natural disease course and it should therefore be considered a palliative treatment [37].

ECT may also be used as a part of a more integrated approach with other treatment modalities, such as immunotherapy. An abscopal effect has been reported in patients treated with ipilimumab and radiotherapy (RT), in which radiation of a tumour causes regression of untreated distant skin lesions [38]. Although not well understood, it has been proposed that this is an immune-mediated phenomenon, suggesting that immunotherapy and RT could have potentially synergistic effects. Similarly, it is possible that immune checkpoint blockade might also be able to improve the effects of ECT. In a retrospective analysis of 15 patients with previously treated metastatic melanoma who received ipilimumab with ECT, a local ORR was observed in 67% of patients [39]. In a comparison of ipilimumab alone versus ipilimumab plus local peripheral treatments (i.e. radiotherapy or electrochemotherapy) in 127 melanoma patients, the addition of local treatment significantly prolonged OS without increased toxicities [40]. ECT in combination with immunotherapy appears to be feasible, tolerable and associated with potent anti-tumour activity and high response rates. However, the optimal timing of ECT in combination with immunotherapy needs further investigation.

### Adjuvant therapy of melanoma

Until recently, IFN-α was the only approved drug for the adjuvant therapy of patients with melanoma at high-risk of recurrence after surgical resection. However, the use of IFN-α remains controversial, with ongoing debate over the optimal dose and regimen. A 1-year regimen comprising of a high-dose IV induction phase followed by a subcutaneous maintenance phase has been accepted as standard in the US and elsewhere. However, the 1-year treatment duration, associated toxicity, and uncertainty over survival benefit has limited its utilisation. In the E1697 trial, 4 weeks of IV induction did not improve 5-year OS compared with observation alone and was associated with worse quality of life in patients with intermediate-risk melanoma [41]. Another option, pegylated-IFN, allows a lower once weekly dose and significantly improved recurrence-free survival (RFS) versus observation in the EORTC 18991 trial, although there was no significant difference in OS [42]. However, almost one-third of patients discontinued therapy due to toxic adverse effects. The future role of IFN as adjuvant treatment is unclear, although it may have a role in patients with ulcerated tumours [43].

High-dose ipilimumab was approved for adjuvant treatment of melanoma in the US in 2015. This was largely based on a study of 951 patients who had undergone complete resection of stage III cutaneous melanoma in which ipilimumab 10 mg/kg resulted in a significantly higher 5-year OS rate than placebo, with a 28% risk reduction for death (HR, 0.72; 95.1% confidence interval [CI] 0.58–0.88; p = 0.001) [44]. However, drug-related toxicity was a major concern with 48% of patients receiving ipilimumab discontinuing treatment. Given the dose-dependency of ipilimumab toxicity, the efficacy and safety of lower dose ipilimumab 3 mg/kg as adjuvant therapy is currently been investigated in the E1609 study. In preliminary results, ipilimumab 3 mg/kg showed similar RFS as ipilimumab 10 mg/kg with significantly less toxicity [45].

Several other trials of adjuvant checkpoint inhibitor or targeted therapy are ongoing (Table 2). In the CheckMate-238 trial, ipilimumab 10 mg/kg was compared with nivolumab 3 mg/kg in 906 patients after complete resection of stage IIIB-IV melanoma [46]. RFS at 1 year was 70.5% (95% CI 66.1–74.5) in the nivolumab group compared with 60.8% (95% CI 56.0–65.2) in the ipilimumab group (HR for disease recurrence or death, 0.65; 97.56% CI 0.51–0.83; p < 0.001). The RFS benefit seen with nivolumab was observed in several subgroups, including those categorised by PD-L1 expression, BRAF status or disease stage. Nivolumab was also better tolerated, with fewer treatment-related grade 3–4 adverse events and discontinuations compared with ipilimumab. Based on these results, nivolumab has the potential to be a new standard of care for patients with resected stage IIIB-IV melanoma, regardless of BRAF status. Nivolumab was approved by FDA in the treatment of adjuvant melanoma on December 2017.

**Table 2:** Ongoing adjuvant trials

Study	No. of patients	TNM stage	Therapy	Primary endpoint
US Intergroup E1609	1600	III (IIIB–c), IV (M1a, M1b)	Ipilimumab 3 mg/kg or 10 g/kg vs HD-IFN	RFS, OS
COMBI-AD	852	III (BRAF V600E/K)	Dabrafenib + trametinib vs. placebo	RFS
BRIM-8	725	IIC, III (BRAF V600; Cobas)	Vemurafenib vs. placebo	DFS
EORTC-1325/KEYNOTE-054	900	IIA (> 1 mm met), IIIb–C	Pembrolizumab vs. placebo	RFS, RFS in PDL1+
CheckMate-238	800	IIIB–C, IV	Nivolumab vs. ipilimumab 10 g/kg	RFS
US Intergroup S1404	1240	IIIA (N2), IIIB–C, M	Pembrolizumab vs. HD-IFN or ipilimumab 10 mg/kg	RFS, OS
C heckMate-915	1125	IIIB–D, IV	Ipilimumab + nivolumab vs ipilimumab or nivolumab	RFS

Adjuvant use of targeted agents has also been reported. In the BRIM-8 trial, 1 year of adjuvant monotherapy with the BRAF inhibitor vemurafenib provided a significant improvement in disease-free survival (DFS) (46% risk reduction, p = 0.0010) compared with placebo in 314 patients with resected stage IIC, IIIA or IIIB BRAF-mutated melanoma [47]. However, in a cohort of 184 patients with stage IIIC BRAF-mutated melanoma, the increase in median DFS with adjuvant vemurafenib was not significant (23.1 vs 15.4 months; HR 0.80, 95% CI 0.54–1.18; p = 0.2598). Treatment was generally well tolerated, with no increase in secondary skin cancers [cutaneous squamous cell carcinoma (SCC) and keratoacanthoma (KA)] known to be associated with vemurafenib.

BRAF inhibition has also been assessed in combination with MEK inhibition. In the Combi-AD trial of adjuvant dabrafenib plus trametinib in stage III BRAF-mutated melanoma, estimated 3-year RFS was 58% with combination therapy (n = 438) versus 39% with placebo (n = 432) (HR for relapse or death, 0.47; 95% CI 0.39–0.58; p < 0.001) [48]. Three-year OS was 86% with the combination compared with 77% in the placebo group (HR for death, 0.57; 95% CI 0.42–0.79; p = 0.0006). However, 41% of combination-treated patients had grade 3–4 adverse events and 26% discontinued treatment because of toxic effects.

In summary, use of IFN as adjuvant therapy can be considered to be in its ‘retirement’ phase. High-dose ipilimumab is approved in the US but anti-PD-1 agents appear to be more effective and better tolerated, while combined BRAF and MEK inhibition may have a role in BRAF-mutated melanoma. The debate over whether to choose immunotherapy or targeted therapy has now extended to the adjuvant setting.

## Conclusions

Advances in the treatment of advanced melanoma have significantly improved the long-term prognosis for patients in recent years. While treatment was previously generally considered as being of palliative intent, the development of novel immunotherapies and targeted agents has completely altered the therapeutic landscape. Treatment with these agents has resulted in survival outcomes significantly improved as compared to what was previously achieved with other treatment modalities such as chemotherapy. The focus is now on achieving greater treatment efficacy through the use of various combination approaches, including combinations of different immunotherapies as well as with non-immunotherapeutic options, in particular the new targeted therapies. Doublet and even triplet combinations involving these new agents will likely become new gold standards of care. However, more research to identify the optimal combination and sequencing of treatments is needed. Moreover, in order to maximise the benefits of these various new treatment options, selection of the most appropriate patients for therapy is essential. Various candidate biomarkers [e.g. PD-L1 expression, CD8^+^ TILs, tumour mutational burden (TMB), neoantigen burden, transcriptomic profiling, blood-based biomarkers] are being investigated to help identify patients who most likely will benefit and to assist with increasingly complex treatment decision-making. The development of biomarkers remains a focus of the personalized medicine approaches to guide the use of novel therapeutic strategies to the most appropriate patients.
